# Silencing of FTX suppresses pancreatic cancer cell proliferation and invasion by upregulating miR-513b-5p

**DOI:** 10.1186/s12885-021-07975-6

**Published:** 2021-03-18

**Authors:** Shan Li, Qian Zhang, Wen Liu, Chunbo Zhao

**Affiliations:** Department of Gastrointestinal Radiation Oncology, Cancer Hospital of Harbin Medical University, 150 Haping Road, Nangang District, Harbin City, Heilongjiang Province 150081 P. R. China

**Keywords:** Pancreatic cancer, Long non-coding RNA FTX, miR-513b-5p, Xenotransplantation mouse model

## Abstract

**Background:**

Abnormal expression of long non-coding RNA (lncRNA) FTX (five prime to Xist), which is involved in X chromosome inactivation, has been reported in various tumors. However, the effect of FTX on the development of pancreatic cancer (PC) has not been elucidated. The purpose of this study was to explore the possible molecular mechanism of FTX in PC.

**Methods:**

Quantitative real-time PCR (qRT-PCR) was used to measure the expression levels of FTX and miR-513b-5p in PC cell lines. Proliferation and apoptosis of PC cells were determined by CCK-8, Edu assay, and flow cytometry. Invasion and migration ability of PC cells were detected by Transwell assay and scratch test. Bioinformatics analysis, luciferase reporter gene assay, and RNA immunoprecipitation (RIP) assay were used to verify the direct binding between FTX and miR-513b-5p. The xenotransplantation mouse model was established to explore the effect of FTX and miR-513b-5p on the PC tumor growth in vivo.

**Results:**

The expression levels of FTX were increased in PC cell lines, and silencing of FTX remarkably suppressed the invasion ability and cell viability. Besides, FTX could bind to miR-513b-5p as a competitive endogenous RNA, thus promoting the invasion and proliferation ability of PC cells. Moreover, knockdown of FTX inhibited the tumor growth and increased the expression levels of miR-513b-5p and apoptosis-related proteins in vivo.

**Conclusions:**

FTX could directly combine with miR-513b-5p as a competitive endogenous RNA, thus promoting the occurrence and development of PC in vitro and in vivo.

**Supplementary Information:**

The online version contains supplementary material available at 10.1186/s12885-021-07975-6.

## Background

Pancreatic cancer (PC) is a malignancy of the digestive tract, which is characterized by high mortality rate, poor prognosis, and extremely low cure rate [1–4]. Currently, the main treatment for PC is traditional surgery, radiotherapy and chemotherapy [5]. When clinical symptoms appear, it is often in the middle or late stage, and the 5-year survival rate of patients is less than 5% [6]. Invasion and metastasis are important biological characteristics of PC and the main reasons for poor prognosis of PC patients [7]. However, due to the inconspicuous early symptoms and the lack of effective biomarkers for early diagnosis, most PC patients have shown local invasion or even distant metastasis when seeking treatment, thus missing the best time for treatment [8, 9]. Extensive studies have identified various genes and proteins involved in the regulation of PC invasion and metastasis [10, 11], and the expression and activation of these genes and proteins are regulated by a variety of molecular pathways [12]. However, the pathogenesis of PC remains unclear. Therefore, the key to improve the prognosis of PC is to further study the biological characteristics of PC and the molecular regulatory mechanism of its occurrence and development, and to search for reliable markers for early diagnosis and prognosis evaluation.

The development of molecular biology provides new strategies for the early diagnosis and treatment of PC [13–15]. Long non-coding RNAs (lncRNAs) are non-coding RNAs with a length of more than 200 nucleotides and play important regulatory roles in the physiological and pathological processes of the body through epigenetic, transcriptional, and post-transcriptional regulations [16]. LncRNAs are abnormally expressed in various tumor tissues showing tissue specificity, making them ideal diagnostic markers and potential therapeutic targets [17, 18]. Studies have shown that a variety of lncRNAs played important regulatory roles in the invasion and metastasis of PC [19, 20]. For example, HOXA terminal transcriptional antisense RNA (HOTTIP) is highly expressed in pancreatic ductal adenocarcinoma, and silencing of HOTTIP can suppress the growth and metastasis of PC cells and promote the apoptosis of cancer cells [21]. LncRNA-ROR can trigger the occurrence of epithelial-mesenchymal transition (EMT) and promote the invasion and metastasis of PC [22]. LncRNA-MALAT can maintain the stability of PC stem cells by upregulating SOX2 [19]. Studies have found that lncRNA FTX (five prime to Xist) was abnormally expressed in various cancers, such as hepatocellular carcinoma, renal cell carcinoma and colorectal cancer [23–25], but the regulation of lncRNA FTX in PC remains unclear.

MicroRNAs (miRNAs) are non-coding miRNAs with about 18–25 nucleotide in length and can inhibit the expression of target mRNAs by targeting their 3′-UTR region [26]. Altered expression of miRNAs have been shown to play important roles in the invasion and metastasis of PC [27]. It was reported that miR-513b-5p had low expression levels in gastric cancer, lung cancer and colon cancer, and acted as an oncogene [28–30]. However, the expression of lncRNA FTX in PC and its biological mechanism are unclear.

In this study, the expression levels of lncRNA FTX in PC cell lines were detected, and appropriate PC cells were selected to verify the interaction between lncRNA FTX and miR-513b-5p. In addition, changes in the biological behaviors of PC cells such as proliferation, migration, invasion and apoptosis were detected by inhibiting the expression of lncRNA FTX. The aim of this study was to explore the role of FTX and miR-513b-5p in PC and their possible mechanisms, hoping to provide insights into the early diagnosis and treatment of PC.

## Methods

### Cell culture

HPDE6-C7, HS-766 T, SW1990, AsPC-1P, BxPC-3, and PANC-1 cells were purchased from ATCC (Manassas, VA). RPMI 1640 medium (GIBCO, Carlsbad, CA) supplemented with 10% Fetal Bovine Serum (FBS, Gibco, Thermo Fisher Scientific) and 1% streptomycin-penicillin (Gibco, Thermo Fisher Scientific) was used to incubate the cells. All cell lines were cultured in a humidified incubator at 37 °C with 5% CO_2_.

### Cell transfection

Negative control (NC) vector and LV-FTX vector were constructed using lenti-pac HIV expression package mixture and lentiviral vector (Gene Copoeia, Rockville, Md) following the manufacturer’s instructions. All cell lines were seeded into 6-well plates and incubated overnight to reach 4 × 10^5^ cells per well. The original culture medium was replaced with 2 ml fresh culture medium containing 6 g/ml polybrene, and appropriate viral suspension was added for incubation at 37 °C. Transfected cells were incubated for 48 h for subsequent experiments.

### Cell proliferation and apoptosis assay

Cell viability was determined by CCK-8 kit (Dojindo, Japan). The proliferation capacity was detected using Cell Light™ EdU kit (RiboBio, Nanjing, China). After pretreatment, 50 μM EdU was added to the medium to culture cells for another 2 h. After fixed with 4% paraformaldehyde, the cells were staining with Hoechst 33342 (1:10,000, Sigma, USA) at room temperature for 10 min. EdU positive cells (1 × 10^5^) were counted by fluorescence microscope (Nikon, Tokyo, Japan). Moreover, cell apoptosis was measured using Annexin V-FITC/PI kit (KeyGEN, Nanjing, China) for 15 min in dark. The Negative control (NC) and LV-FTX cells (1 × 10^5^) were washed twice with PBS and re-suspended in PBS, then stained with Annexin V (1:1000) and PI for 15 min in dark. Apoptosis rates were analyzed using FACSAria flow cytometry (BD Biosciences, Franklin Lakes, NJ).

### Cell cycle detection

Cells were inoculated into a 6-well plate with a cell density of 1 × 10^6^ cells per well. After 24 h, the cells were digested by trypsin and fine cells in the supernatant were collected and washed with PBS for 3 times. Cells were then fixed with 70% ethanol, and each cell sample was resuspended and added with 500 L pre-mixed PI dye. Cells were resuspended and incubated in dark for 30 min. GO/G1 cell rations were analyzed using FACSAria flow cytometry (BD Biosciences, Franklin Lakes, NJ).

### Cell migration and invasion assay

For cell migration assay, all cell lines after transfection were seeded in six-well plates for 48 h to form a confluent monolayer. Then a 10 μl pipette tip was used to scratch cells, and images were captured using a fluorescence microscope (Nikon, Tokyo, Japan) at 0 h and 24 h after the wound. ImageJ software (NIH, Bethesda, MD) was used to analyze wound healing to assess cell migration. For cell invasion assay, cell migration was assessed by the number of cells (1 × 10^6^/ml) passing through a polycarbonate membrane (8 μm well, BD Biosciences, San Jose, CA, USA). In addition, the treated cell suspension was added to the upper chamber of Transwell (Costar, Badhoevedorp, Netherlands) covered with artificial basement membrane, and RPMI 1640 medium containing 10% Fetal Bovine Serum (FBS, Gibco, Thermo Fisher Scientific) and 1% streptomycin-penicillin (Gibco, Thermo Fisher Scientific) was added to the lower compartment. Cells were cultured at 37 °C for 24 h, followed by stained with 5% crystal violet solution and counted under the fluorescence microscope (Nikon, Tokyo, Japan) to evaluate the invasion ability of the cells.

### qRT-PCR and Western blot

qRT-PCR was performed as described in literature [31]. GAPDH and U6 were used as the internal references. The expression levels of FTX and miR-513b-5p were calculated using 2^-ΔΔCt^ method. The primer sequences were listed as below:

- FTX forward 5′-CAAAGCTGGTCCTGTGCCTG-3′
- FTX reverse 5′-ATTGAGTGTGGCATCACCTCC-3′
- GAPDH forward, 5′-GGGCTGCTTTTAACTCTGGT-3′
- GAPDH reverse, 5′-GCAGGTTTTTCTAGACGG-3′
- miR-513b-5p forward; 5′-GGCCGGGGAGCTGGAGAAGA-3
- miR-513b-5p reverse; 5′-TCCATGGAGGGTTGGGGGTTCC-3′
- U6 forward, 5′-CTCGCTTCGGCAGCACATATATT-3′
- U6 reverse, 5′-ACGCTTCACGAATTTGCGTGGC-3′.

Western Blot analysis was performed as described in literature [32]. Total proteins were extracted from cells and tissues. BCA method was used to detect the concentration of total proteins. Protein samples were electrophoretic separated by SDS-PAGE gel and transferred to PVDF membrane. Next, BSA was sealed at room temperature for 2 h, and Cyclin D1 (ab16663), PCNA (ab29), c-caspase-3 (ab49822) and c-caspase-12 (ab62463) were incubated at 4 °C with shaking overnight. Secondary antibodies were then added and incubated at room temperature for 2 h. TBST was cleaned for 3 times. Luminescent solution was used for exposure. GAPDH (ab8245) were used as the internal reference, and the relative expression levels of protein were quantitatively analyzed by ImageJ software.

### Luciferase activity and RIP assay

For detection of luciferase activity, PCR was performed to amplify the partial sequences of FTX which contained the putative binding sites of miR-513b-5p. The sequences were then cloned into the pmirGLO Dual-Luciferase miRNA Target Expression Vector (Promega Corp., Fitchburg, WI, USA). GeneArt™ Site-Directed Mutagenesis system (Thermo Fisher Scientific, Inc.) was used to induce site-directed mutagenesis of miR-513b-5p complementary bases in the sequence of FTX. Then, miR-513 mimic or its control was co-transfected with FTX-WT or FTX-MUT, and luciferase activity in the cells was detected using the dual-luciferase Reporter Assay System (Promega, Madison, WI, USA) 24 h post-transfection. Immunoprecipitation (RIP) was detected using the Magna RIP™ RNA binding protein immunoprecipitation kit (Millipore, Bedford, MA, USA).

### Establishment of mouse xenotransplantation model

Twelve BALB/c nude mice (female, 8 weeks old, obtained from Shanghai animal experimental center) were used to establish the xenotransplantation model. Briefly, tumor volume was measured every 5 d after subcutaneous injection of 100 μl AsPC-1 (1 × 10^6^ cells) suspension (transfected with LV-NC or LV-FTX) into the left abdomen of mice. Mice were anesthetized with 3% sodium pentobarbital (30 mg/kg body weight, Sigma, USA) through intraperitoneal injection and sacrificed by cervical dislocation after 30 d. Animal experiments were conducted in accordance with the Cancer Hospital of Harbin Medical University guidelines for the care and use of experimental animals strictly. All experiments were approved by the Ethics Committee of Cancer Hospital of Harbin Medical University.

### Immuno-histochemical staining (IHC) analysis

The separated PC tumor tissues were embedded in paraffin and sectioned for IHC test. In brief, the tissue sections were incubated with 0.15% Triton X-100, and then were closed with 1% goat serum albumin (Flarebio, Wuhan, China). The sections were then incubated with Ki-67 antibody (Abcam, Cambridge, UK) at a dilution of 1:500. Next, the sections were incubated with secondary antibody (1:5000 Abcam, Cambridge, UK) at 37 °C for 30 min.

### Statistical analysis

All experiments were performed in three independent experiments, and data were expressed as mean ± standard error (SE). GraphPad Prism 6.0 (GraphPad Software Inc., La Jolla, CA, USA) was used to analyze the data. ANOVA and Student’s t test were used for significance analysis. *P* < 0.05 was considered statistically significant.

## Results

### The expression of FTX and miR-513b-5p in PC cell lines

**Fig. 1:**
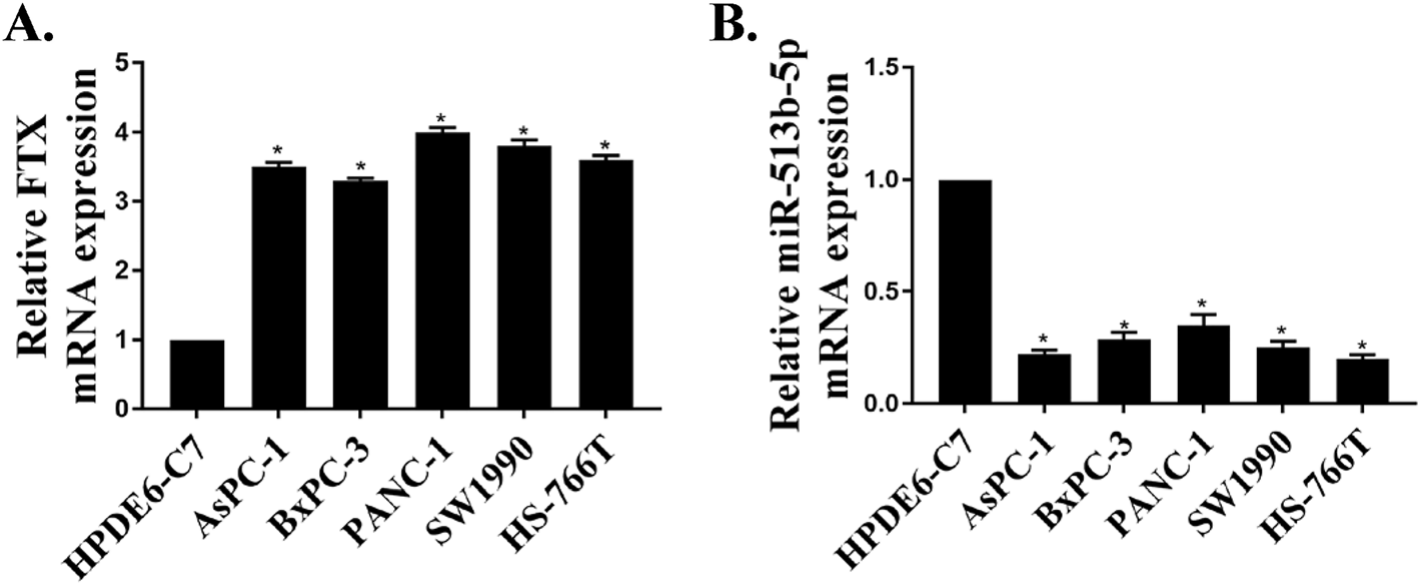
Expression of FTX and miR-513b-5p in PC cell lines. Detection of the FTX (**a**) and miR-513b-5p (**b**) expression in PC cell lines (HPDE6-C7, AsPC-1, BxPC-3, PANC-1, SW1990 and HS-766 T) by qRT-PCR. * *P* < 0.05

The expressions of FTX and miR-513b-5p in PC cell lines were determined by qRT-PCR. As shown in Fig. 1a, compared with HPDE6-C7 cells, FTX was remarkably up-regulated in PC cell lines (*P* < 0.05). Furthermore, compared with the HPDE6-C7 cells, the expression levels of miR-513b-5p in PC cell lines were lower (Fig. 1b, *P* < 0.05). qRT-PCR results showed that FTX was significantly upregulated, whereas miR-513b-5p had a lower expression levels in PC cells compared with that in HPDE6-C7 cells. These results indicated that FTX and miR-513b-5p might be related to the development of PC.

### Effects of silencing of FTX on proliferation, apoptosis and cell cycle of PC cells

**Fig. 2:**
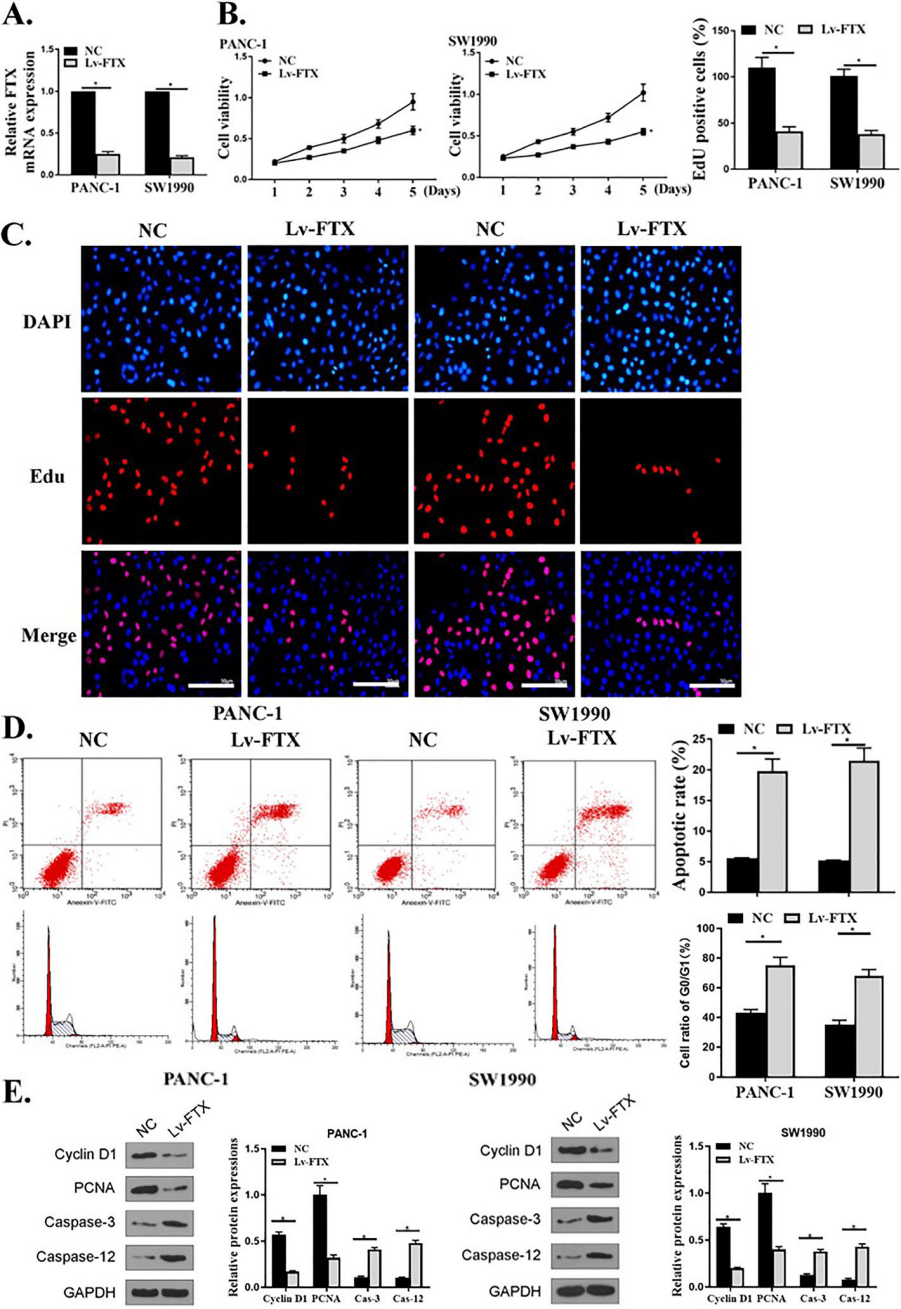
Effects of silencing of FTX on proliferation and apoptosis of PC cells. **a** Measurement of the expression of FTX in PANC-1 and SW1990 cells by qRT-PCR. **b**, **c** Measurement of the proliferation activity of PANC-1 and SW1990 cells transfected with LV-FTX by CCK8 (**b**) and EDU (**c**) assays with EdU (red) and Hoechst 33342 (blue), compared with the control group. **d** Measurement of the apoptosis rates and cell cycle of PANC-1 and SW1990 cells between LV-FTX group and control group by flow cytometry. **e** Measurement of the protein expression of Cyclin D1, PCNA, cleaved caspase-3 (c-caspase-3) and cleaved caspase-12(c-caspase-12) in PANC-1 and SW1990 cells of LV-FTX and the control group by Western Blot. * *P* < 0.05

To explore the effect of FTX on the biological function of PC cells, LV-FTX was transfected into PANC-1 and SW1990 cells to evaluate the effects of silencing of FTX on the proliferation and apoptosis of PC cells. As shown in Fig. 2a, the expression levels of FTX were markedly decreased in PANC-1 and SW1990 cells transfected with LV-FTX (*P* < 0.05), indicating that FTX was successfully silenced by LV-FTX transfection in PC cells. After successful transfection, the effects of silencing of FTX on the cell viability and proliferation ability were assessed. It showed that the cell viability and Edu positive cell number of PANC-1 and SW1990 cells in LV-FTX group were observably reduced compared with that in the negative control group (*P* < 0.05) (Fig. 2b and c), suggesting that cell viability and proliferation ability were significantly suppressed after silencing of FTX. Furthermore, flow cytometry and western blot were conducted to examine the effect of silencing of FTX on PC cells. The results showed that the apoptosis rates of PANC-1 and SW1990 cells in LV-FTX group were remarkably higher compared to that in the control group (Fig. 2d, *P* < 0.05), indicating that mass apoptosis cells appeared in the LV-FTX transfection group by silencing of FTX. Meanwhile, Western Blot results showed that compared with the control group, the expression levels of cleaved caspase 3 (c-caspase-3) and cleaved caspase 12 (c-caspase-12) were markedly increased in PANC-1 and SW1990 cells of LV-FTX group (Fig. 2e, *P* < 0.05). As c-caspase-3 and c-caspase-12 are family members of caspases that are critical mediators of programmed cell death [33], suggesting that the probability of apoptosis was greatly increased by silencing of FTX. These results demonstrated that silencing of FTX could significantly suppress the proliferation and promote apoptosis of PC cells. Moreover, the ration of G0 and G1 cells was detected by flow cytometry and the expression of Cyclin D1 and PCNA were determined by western blot. It showed that silencing of FTX induced PC cell cycle arrest at G0/G1 phase (Fig. 2d, *P* < 0.05) and decreased the expression levels of Cyclin D1 and PCNA (Fig. 2e, *P* < 0.05). We therefore concluded that silencing of FTX may inhibit cell proliferation and promote apoptosis by regulating cell cycle.

### Effects of silencing of FTX on invasion and migration of PC cells

**Fig. 3:**
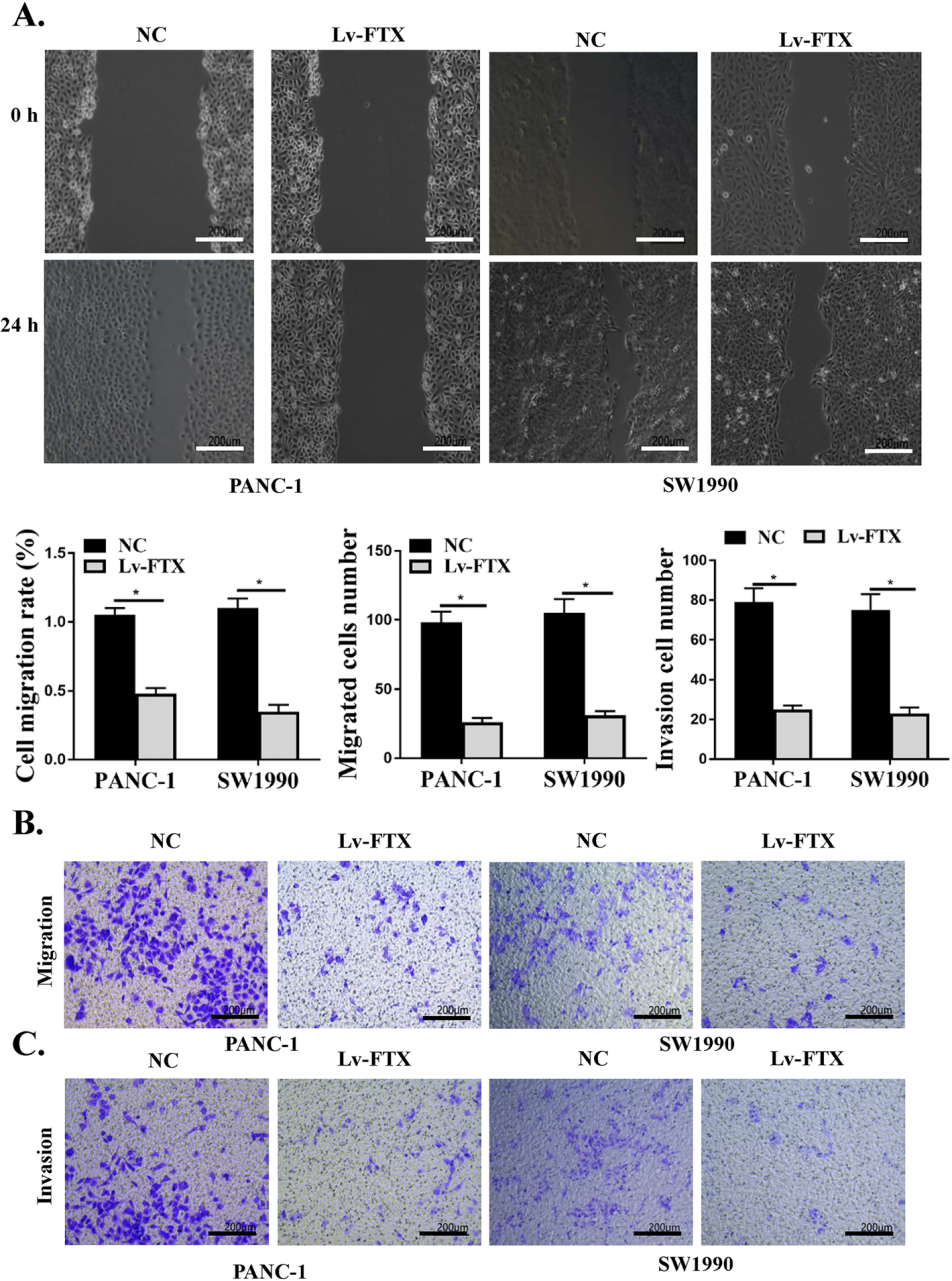
Effects of silencing of FTX on migration and invasion of PC cells. **a** Detection of the PANC-1 and SW1990 cells in LV-FTX and control group mobility by wound healing assay. **b**, **c** Detection of the ability of cell migration (**b**) and invasion (**c**) by Transwell assay. * *P* < 0.05

In addition, to explore whether silencing of FTX could impact the invasion and migration of PC cells, the migration ability of cells was detected by wound-healing assay, and the results showed that compared with the control group, the migration rates of PANC-1 and SW1990 cells with the silencing of FTX were observably decreased after 24 h wound (Fig. 3a, *P* < 0.05). The invasion and migration ability of PC cells were measured by Transwell assay. It showed that the invasion and migration numbers of PANC-1 and SW1990 cells with the silencing of FTX were remarkably decreased compared with the control group (Fig. 3b and c, *P* < 0.05). These results demonstrated that silencing of FTX suppressed the pathogenesis of PC by inhibiting the migration and invasion of PC cells.

### Identification of miR-513b-5p as the target of FTX

**Fig. 4:**
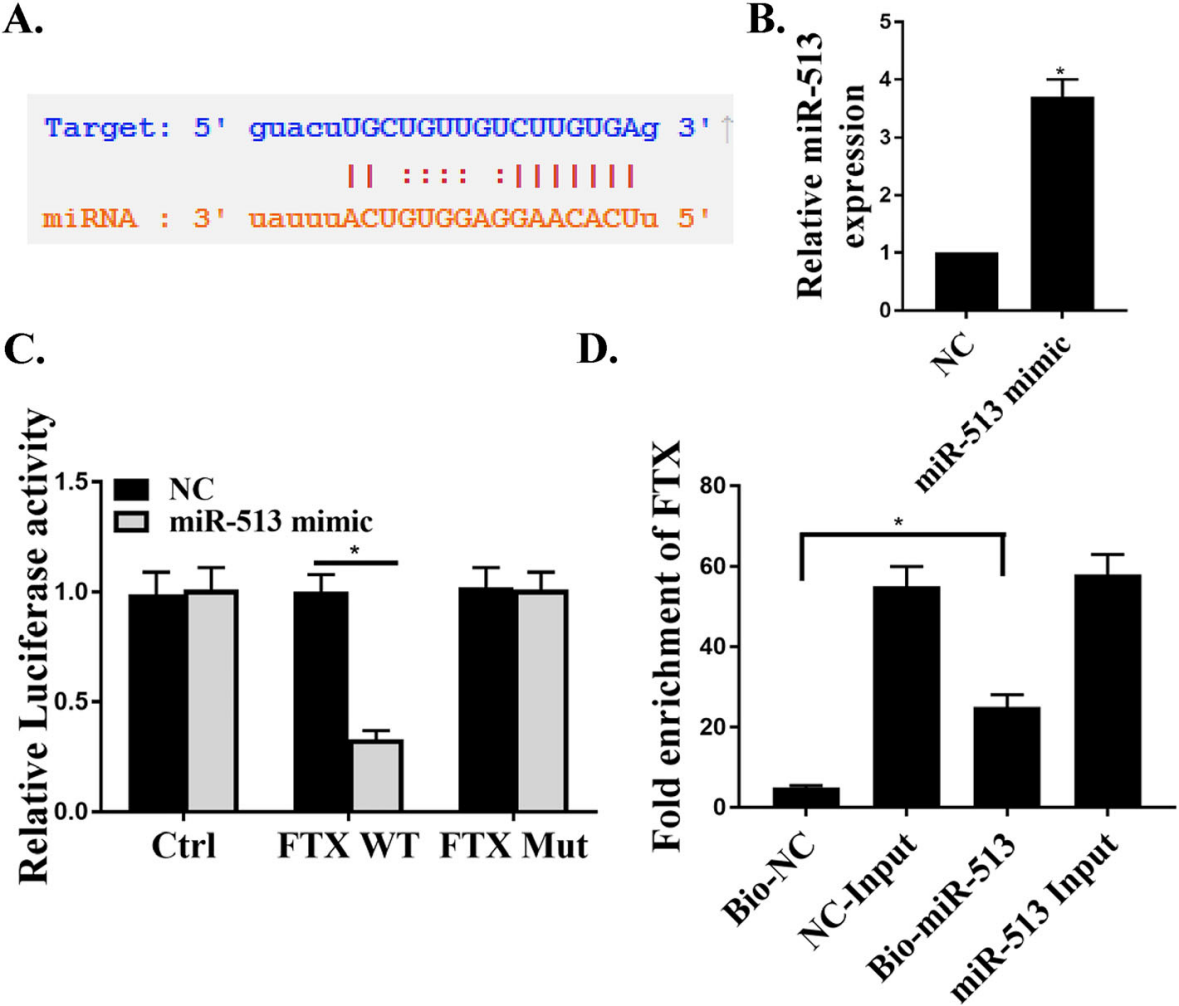
Identification of miR-513b-5p as the target of FTX. **a** Schematic diagram of potential binding sites between miR-513-5p and FTX. **b** Determination of miR-513 expression in transfected miR-513b-5p mimic and negative control cells. **c** Detection of luciferase activity of transfected miR-513b-5p mimic group and negative control cells in control, FTX-WT or FTX-MUT cells. **d** Detection of FTX expression by RIP and qRT-PCR. * *P* < 0.05

It is known that lncRNAs can interact with miRNAs and act as competing endogenous RNAs [34]. Therefore, we speculated that FTX might interact with miRNAs in PC cells. Firstly, the potential binding site of FTX in miR-513b-5p was predicted based on bioinformatics analysis using starBase v2.0 software (http://starbase.sysu.edu.cn/) (Fig. 4a). In addition, the transfected miR-513b-5p mimic observably increased the expression levels of miR-513b-5p (Fig. 4b, *P* < 0.05), indicating that miR-513b-5p mimic was successfully transfected. Then, the direct binding between FTX and miR-513b-5p was verified by luciferin gene reporter assay. Compared to the control group, miR-513b-5p mimic significantly decreased the luciferase activity in FTX-WT cells (Fig. 4c, *P* < 0.05), but had no significant effect on FTX-MUT cells (*P* > 0.05), indicating that FTX could bind with miR-513b-5p to reduce luciferase activity in FTX-WT cells. Meanwhile, RIP results further verified the direct binding between FTX and miR-513b-5p. As shown in Fig. 4d, compared to the control group, Bio-miR-513 caused FTX to raise, indicating that mir-315 could bind to FTX. These results confirmed that miR-513b-5p was a direct target of FTX.

### Effects of miR-513b-5p on PC cell proliferation and invasion

**Fig. 5:**
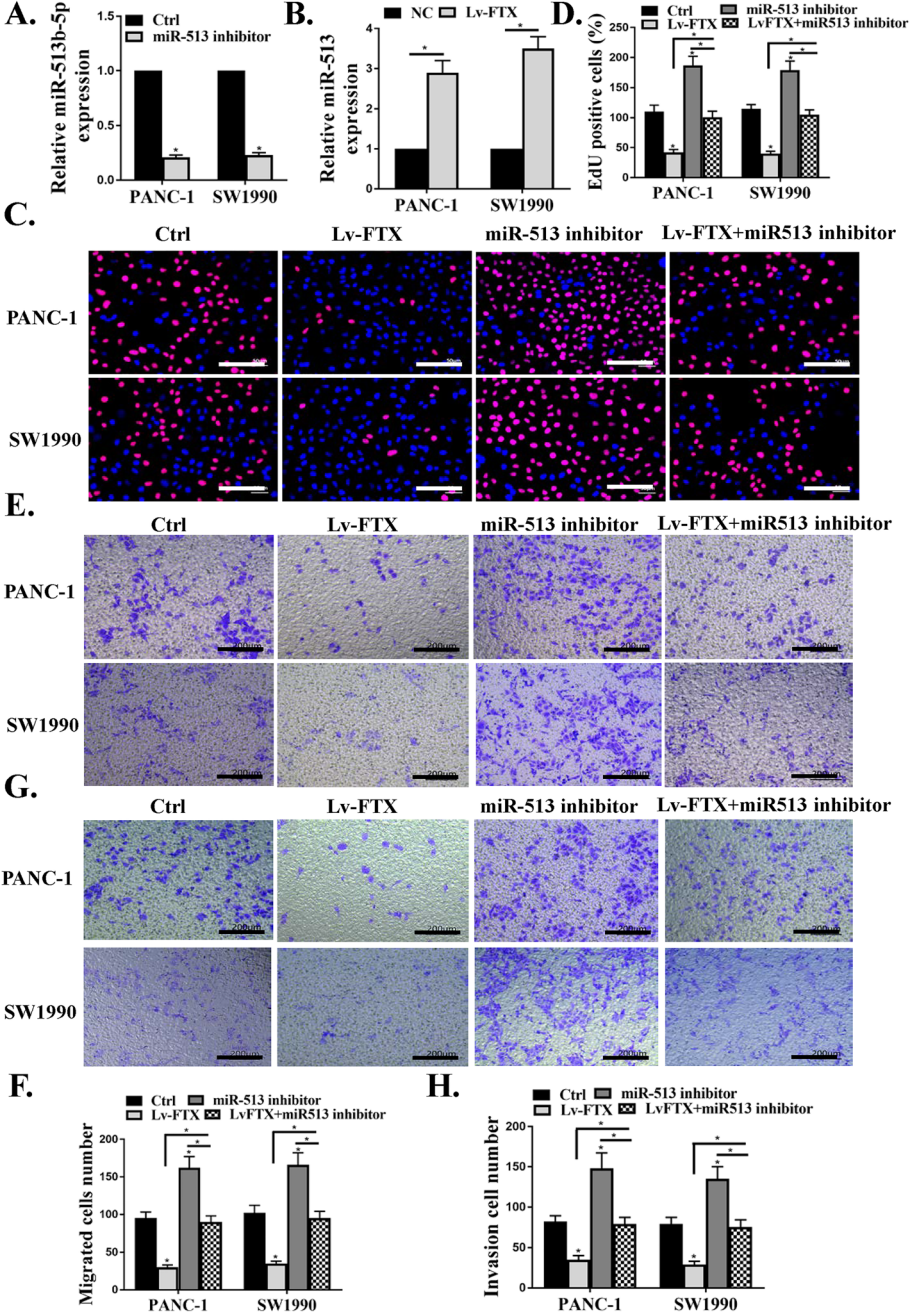
Effects of miR-513b-5p on PC cell proliferation and invasion. **a** Effect of miR-513b-5p inhibitor on the expression of miR-513b-5p in PANC-1 and SW1990 cells. **b** Effect of silencing of FTX on the expression of miR-513b-5p in PANC-1 and SW1990 cells. **c**, **d** Measurement of the proliferation activity of PANC-1 and SW1990 cells by Edu assay with EdU (red) and Hoechst 33342 (blue). **e**, **f** Determination of the migration ability of PANC-1 and SW1990 cells. (G, H) Determination of the invasion ability of PANC-1 and SW1990 cells. * *P* < 0.05

To explore the effect of miR-513b-5p on cell behaviors, PANC-1 and SW1990 cells were treated with miR-513b-5p inhibitor. Compared with the control group, miR-513b-5p inhibitor observably inhibited the expression of miR-513b-5p in PANC-1 and SW1990 cells (Fig. 5a, *P* < 0.05), confirming that miR-513b-5p inhibitor could effectively suppress miR-513b-5p. In addition, the expression levels of miR-513b-5p in PANC-1 and SW1990 cells of LV-FTX groups were significantly higher than that in the control group (Fig. 5b, *P* < 0.05), suggesting that silencing of FTX remarkably increased the expression levels of miR-513b-5p in PC cells. Furthermore, Edu assay showed that miR-513b-5p inhibitor observably increased the number of Edu-positive cells in PANC-1 and SW1990 cells (Fig. 5c and d, *P* < 0.05), while silencing of FTX remarkably reversed this promotion (*P* < 0.05). Moreover, Transwell assay showed that miR-513b-5p inhibitor remarkably promoted the invasion, migration (Fig. 5e and f, *P* < 0.05) and invasion (Fig. 5g and h, *P* < 0.05) of PANC-1 and SW1990 cells, while silencing of FTX showed the opposite effect. These results demonstrated that silencing of FTX could effectively inhibit the proliferation and invasion of PC cells, whereas this phenomenon could be promoted by miR-513b-5p inhibitor in vitro.

### Effects of FTX on PC tumor growth in vivo

**Fig. 6:**
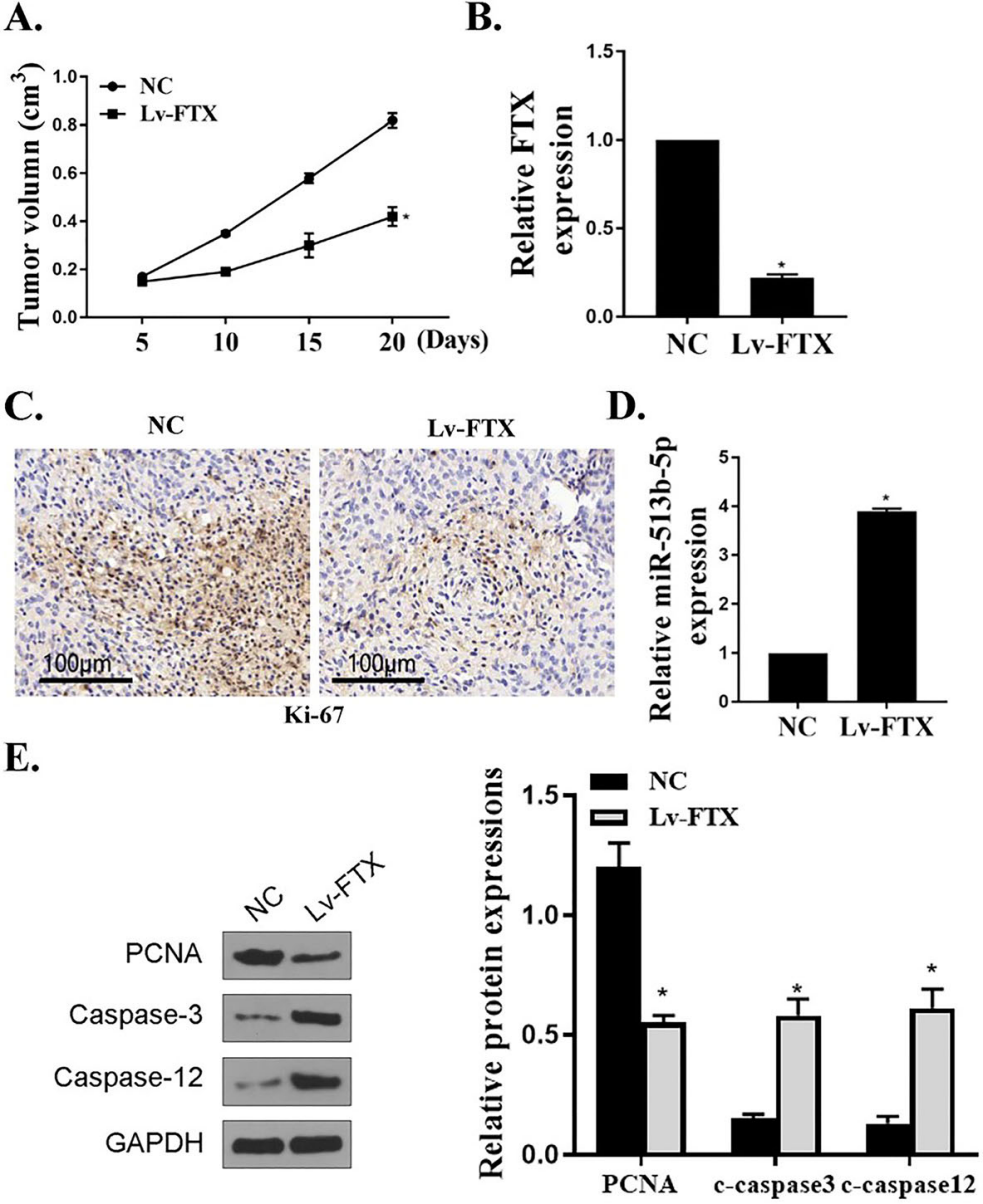
Effects of FTX and miR-513b-5p on PC tumor growth in vivo. **a** Measurement of PC tumor volume in LV-FTX and control group of mice. **b** Determination of the expression of FTX in PC tumors in group of mice. **c** Ki-67 IHC staining in PC tumor tissues of mice. **d** Determination of the expression of miR-513b-5p in PC tumors in mice of group. (E) Determination of the expression levels of PCNA, c-caspase-3 and c-caspase-12 in PC tumor tissues of group by Western Blot. *n* = 6, * *P* < 0.05

The effects of FTX on PC tumor growth in vivo were investigated by establishing xenotransplantation mouse model. Mice were injected with transfected LV-NC or LV-FTX AsPC-1 cells that had a high transfection rate. Compared to the control group, PC tumor volume in LV-FTX group was remarkably decreased (Fig. 6a, *P* < 0.05). In addition, compared to the control group, the expression of FTX in PC tumor tissues of LV-FTX group was observably down-regulated (Fig. 6b, *P* < 0.05), indicating that LV-FTX successfully silenced FTX in PC tumor tissues. In addition, IHC results showed that, compared to the control group, the number of Ki-67 positive cells in PC tumor tissues of LV-FTC group was markedly decreased (Fig. 6c), suggesting that silencing of FTX inhibited the growth of PC cells in vivo. Meanwhile, the expression levels of miR-513b-5p in PC tumor tissues of LV-FTX group was observably higher than that of the control group (Fig. 6d, *P* < 0.05), suggesting that silencing of FTX promoted the expression of miR-513b-5p in PC tumor tissues. Finally, Western Blot results showed that, compared to the control group, the expression of PCNA, c-caspase-3 and c-caspase-12 in PC tumor tissues in LV-FTX group were remarkably up-regulated (Fig. 6e, *P* < 0.05), indicating that silencing of FTX promoted the apoptosis of PC cells and decreased cell proliferation. These results demonstrated that silencing of FTX could suppress the growth of PC tumors in xenotransplantation model mice.

## Discussion

PC affects the digestive system and is one of the malignant tumors [35]. Extensive studies have identified a variety of lncRNAs that play important regulatory roles in the progression of PC [36, 37]. Abnormal expression of lncRNA FTX has been observed in liver cell carcinoma, renal cell carcinoma, colorectal cancer, and other cancers [23, 38]. However, the mechanism of FTX in PC is still unclear.

In this study, the expression of FTX in PC cell lines was determined by qRT-PCR, and it showed that FTX was up-regulated in all 5 PC cell lines, suggesting that FTX might be related to the development of PC. In addition, the expression levels of FTX were the highest in PANC-1 and SW1990 cells, so PANC-1 and SW1990 cells were selected for our follow-up in vitro experiments.

Malignant proliferation of cancer cells is one of the biological characteristics of cancer, and the imbalance of apoptosis is an important factor in the development of tumor [39, 40]. Apoptosis is mainly regulated by death receptor mediated apoptosis pathway, mitochondrial pathway and endoplasmic reticulum pathway [41]. Caspase-3 is the key executing enzyme in these pathways. After receiving the stimulation signal, the cells undergo a series of cascade reactions to activate procaspase-3, cleaved to the active caspase-3, and finally induce apoptosis [42]. Caspase-12 is involved in the regulation of activation pathways in ER stress [43]. In this study, we found that silencing of FTX remarkably inhibited cell activity and Edu positive cell number of PANC-1 and SW1990 cells, and increased the apoptosis rates and the expression levels of c-caspase-3 and c-caspase-12, confirming that silencing of FTX observably inhibited the proliferation activity of PC cells and promoted the apoptosis of cells. Invasion and metastasis are important biological characteristics of PC, and advanced PC has the tendency of high distant metastasis and local invasion [44, 45]. We also observed that silencing of FTX induced PC cell cycle arrest at G0/G1 phase and decreased the expression levels of Cyclin D1 and PCNA. Silencing of FTX markedly suppressed the invasion and migration of SW1990 and PANC-1 cells, suggesting that silencing of FTX accelerated relieving of the symptoms of PC by inhibiting the invasion and migration of PC cells.

LncRNAs also play roles in the development of PC as competitive endogenous RNAs (ceRNAs) [46, 47]. CeRNA is a special manifestation of lncRNA, which can competitively combine with corresponding miRNAs, inhibit the activity of miRNAs, regulate the expression of downstream targeted genes, and thus regulating intracellular pathophysiological changes [48]. Studies have found that lncRNA PVT1 and miR-488 can promote the growth and invasion of PC by regulating ceRNA [36]. In addition, it was reported that FTX was upregulated in gliomas cells and could inhibit the expression of miR-342-3p [49]. One study found that Ftx/miR-545 promoted proliferation and cell cycle progression of HCC cells through activation of the PI3K/Akt pathway [24]. However, another study showed the opposite results and found that FTX could inhibit HCC cell proliferation and activity by binding to MCM2 and miR-374a [50]. In this study, we found that the expression levels of miR-513b-5p were decreased in PC cell lines, and inhibition of miR-513b-5p could remarkably promote the growth and invasion ability of PC cells, suggesting that miR-513b-5p might be related to the development of PC. Meanwhile, we found that miR-513b-5p was the direct target of FTX. Besides, by detecting malignant biological behaviors of PC cells, we showed that FTX could promote the growth, invasion and migration of PC cells by targeting miR-513b-5p.

Xenotransplantation model is an ideal animal model for tumor research [51, 52]. At present, xenografted mouse models have been established for PC, gastric cancer, breast cancer and other tumors, which maintained the pathological and histological characteristics of the original tumor from multiple perspectives such as histopathology and genetics [53–56]. In this study, the effects of FTX on PC tumor growth in vivo were investigated by establishing xenotransplantation mouse model. The results showed that silencing of FTX observably suppressed the growth of PC cells in mice. Meanwhile, silencing of FTX promoted the expression levels of miR-513b-5p in PC tumor tissues, and up-regulated the expression of c-caspase-3 and c-caspase-12 in PC tumor tissues, suggesting that silencing of FTX induces the apoptosis of PC cells in vivo. Combining in vivo and in vitro results, we confirmed that silencing of FTX suppressed pancreatic cancer cell proliferation and invasion by upregulating miR-513b-5p.

## Conclusion

In summary, FTX was upregulated in PC cell lines and could inhibit the apoptosis of PC cells and promote their proliferation, migration and invasion by up-regulating miR-513b-5p. Therefore, silencing of FTX suppressed pancreatic cancer cell proliferation and invasion though upregulating miR-513b-5p. Identifying of the function of FTX could provide a new target and approach for the targeted therapy of PC.

## Supplementary Information

**Additional file 1: Supplementary figure-1**. Original blot images of western blot

## Data Availability

The datasets used and/or analyzed during the current study are available from the corresponding author on reasonable request.

## Abbreviations

lncRNA: Long non-coding RNA
PC: Pancreatic cancer
qRT-PCR: Quantitative real-time PCR
RIP: RNA immunoprecipitation
HOTTIP: HOXA terminal transcriptional antisense RNA
miRNA: MicroRNA
IHC: Immuno-histochemical staining
ceRNA: Competitive endogenous RNA
