# Supplementary figures and images for: Silencing of FTX suppresses pancreatic cancer cell proliferation and invasion by upregulating miR-513b-5p
Source: BMC Cancer. 2021 Mar 18;21:290. doi: 10.1186/s12885-021-07975-6 (PMC7977589; doi:10.1186/s12885-021-07975-6)

**Original image for Figure 2E-PANC-1**

**
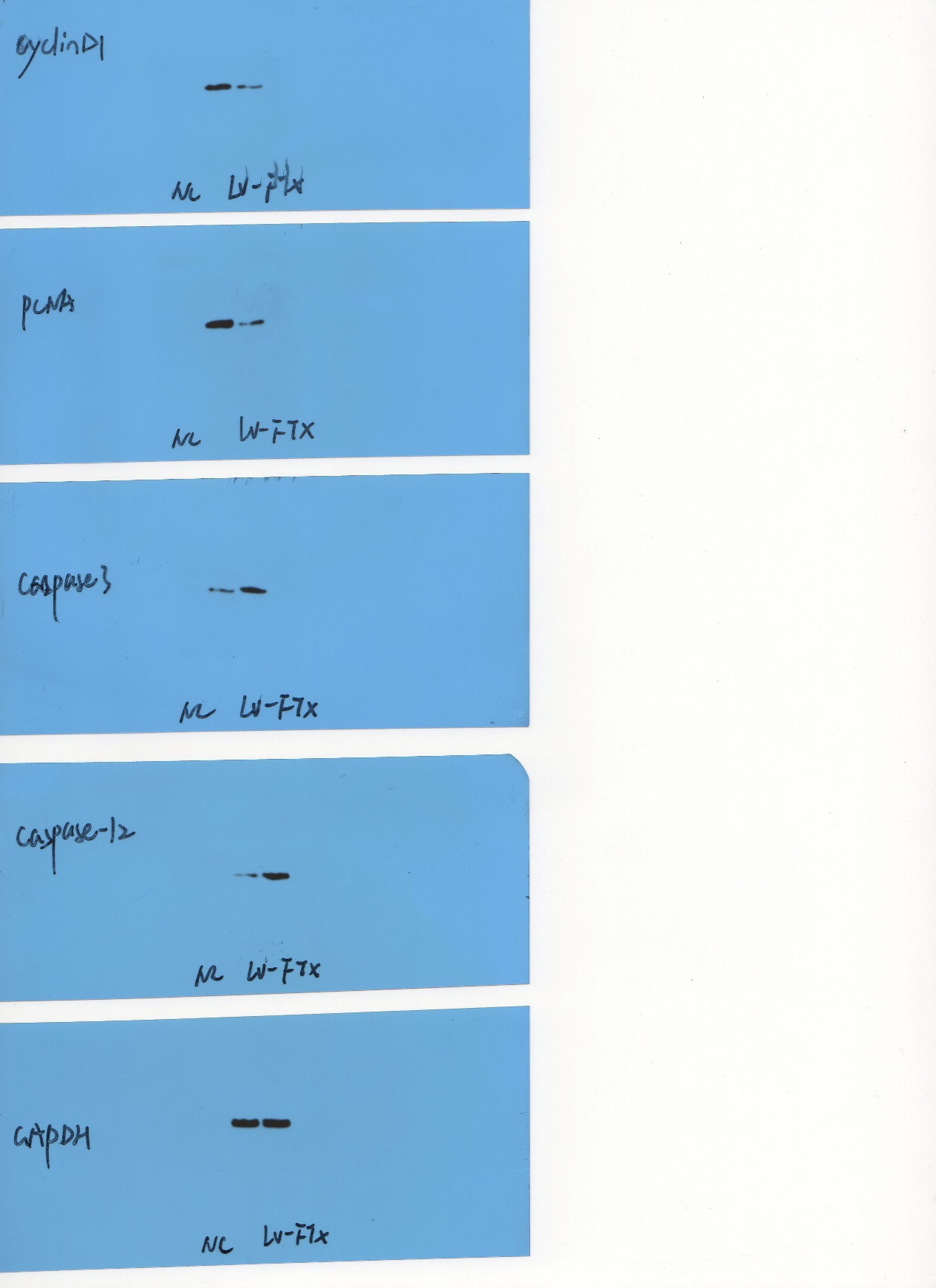
**

**Original image for Figure 2E-SW1990**

**
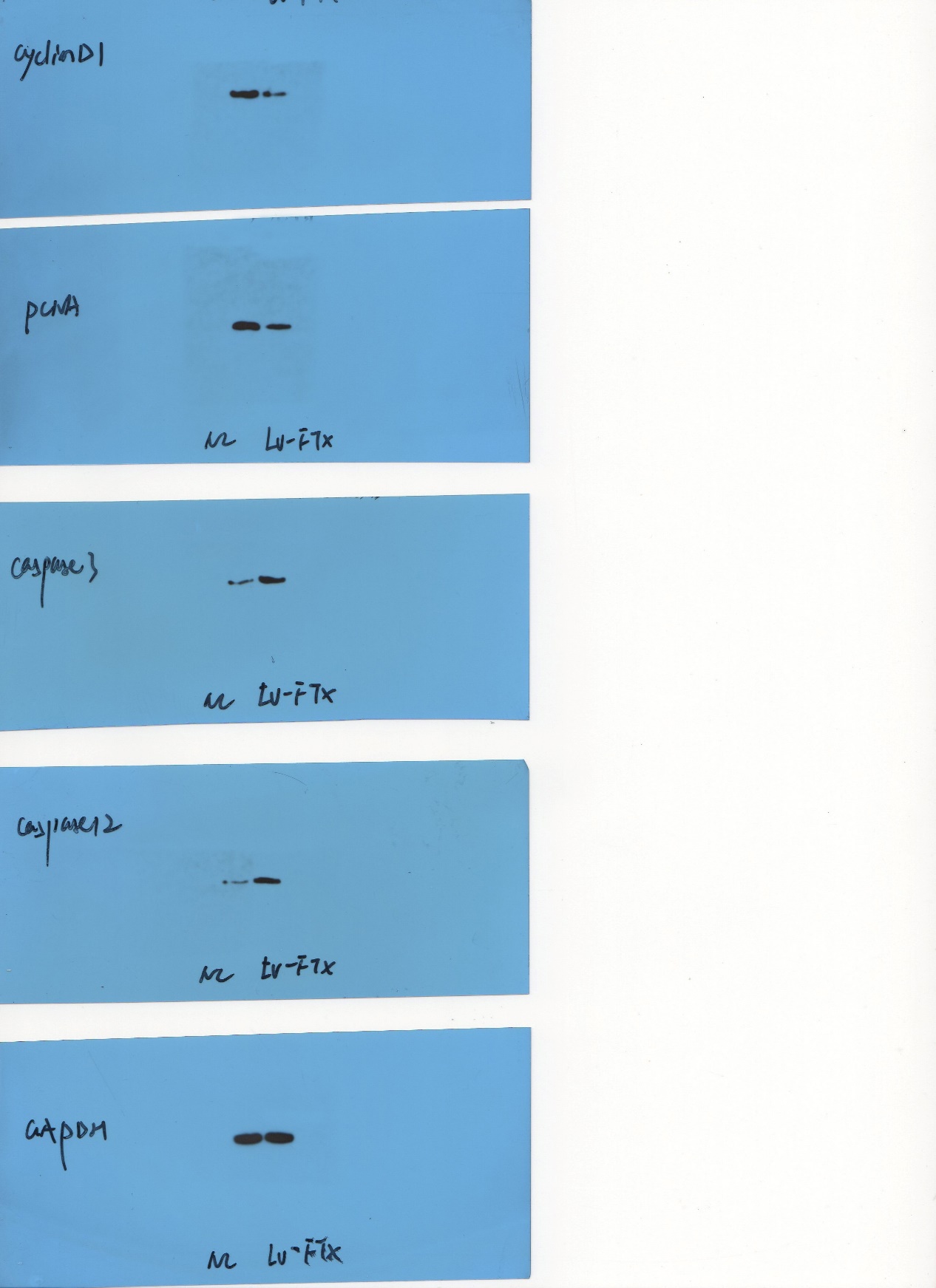
**

**original image for Figure 6E**

**
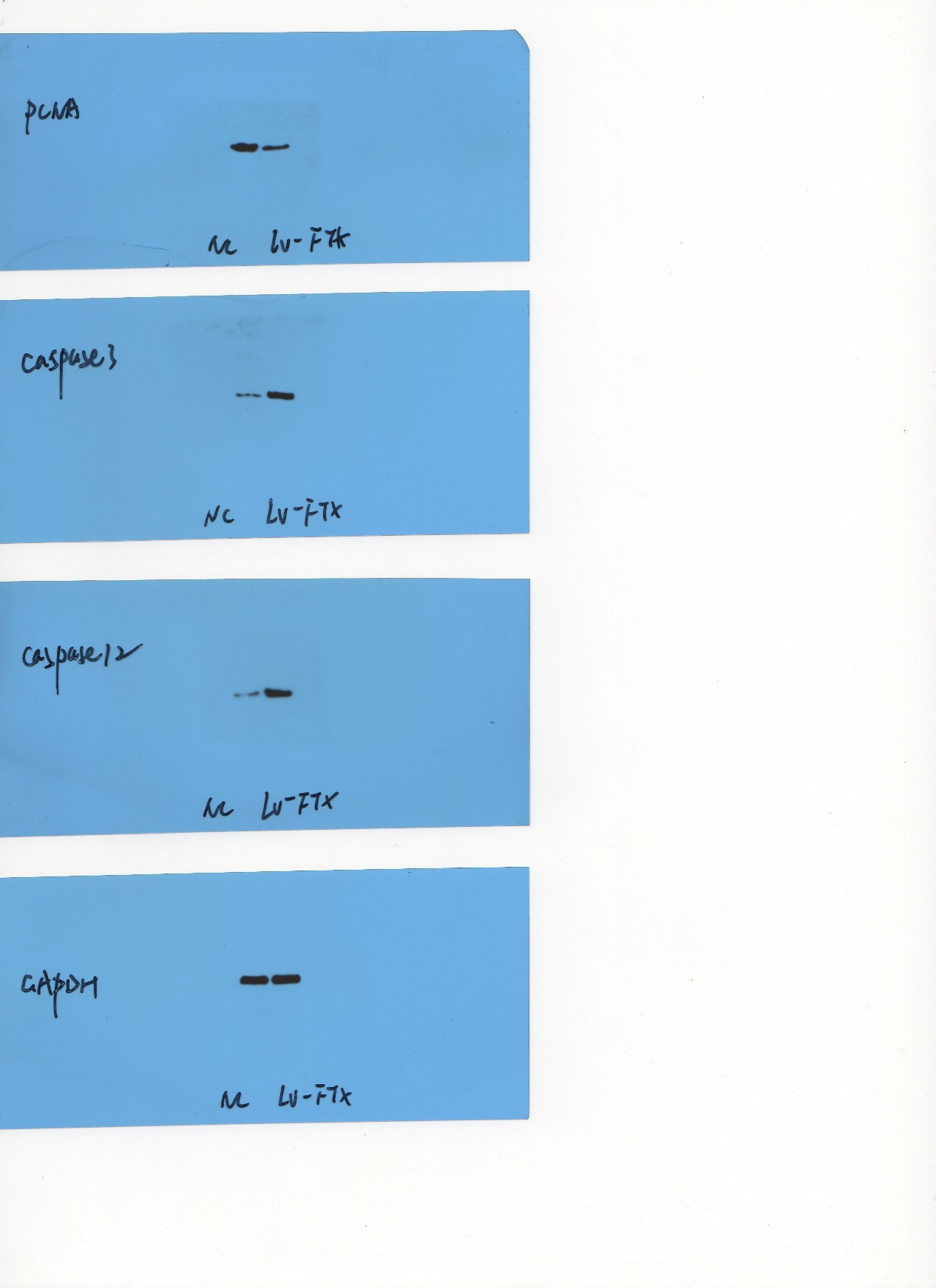
**

Supplement: Supplementary file 1 — Additional file 1: Supplementary figure-1. Original blot images of western blot [file 12885_2021_7975_MOESM1_ESM.docx]
